# Supplementary material for: Room temperature organic exciton–polariton condensate in a lattice
Source: Nat Commun. 2020 Jun 8;11:2863. doi: 10.1038/s41467-020-16656-0 (PMC7280250; doi:10.1038/s41467-020-16656-0)
Supplement: Supplementary file 1 — Supplementary Information [file 41467_2020_16656_MOESM1_ESM.pdf]

Supplementary Information
Room temperature Organic Exciton-Polariton Condensate in a Lattice

M. Dusel[1,†], S. Betzold[1,†], O. A. Egorov[2], S. Klembt[1], J. Ohmer[3], U. Fischer[3], S. Höfling[1,4] & C. Schneider[1,∗]

[1] *Technische Physik, Physikalisches Institut and Würzburg–Dresden Cluster of Excellence ct.qmat, Universität Würzburg, Am Hubland, D-97074 Würzburg, Germany*

[2] *Institute of Condensed Matter Theory and Solid State Optics, Abbe Center of Photonics, Friedrich-Schiller-Universität Jena, Max-Wien-Platz 1, 07743 Jena, Germany*

[3] *Biozentrum, Universität Würzburg, Am Hubland, D-97074 Würzburg, Germany*

[4] *SUPA, School of Physics and Astronomy, University of St Andrews, St Andrews KY16 9SS, United Kingdom*

† *These authors contributed equally to this work*

∗ *Corresponding author. Email: Christian.Schneider@uni-wuerzburg.de (C. S.), Marco.Dusel@uni-wuerzburg.de (M. D.)*

Supplementary Note 1 ”Determination of the exciton-photon coupling strength”

To model the experimentally observed dispersions of polaritons, and to determine the coupling strengths between mCherry excitons and cavity photons, we calculate the eigenstates of a coupled oscillator matrix that accounts for several longitutinal cavity modes which are supported by the device, and which strongly interact with the main excitonic transitions of the mCherry protein. The matrix reads as follows

$$M = \begin{pmatrix} E_{\mathrm{x}} & V & V & V & V \\ V & E_{\mathrm{C1}} & 0 & 0 & 0 \\ V & 0 & E_{\mathrm{C2}} & 0 & 0 \\ V & 0 & 0 & E_{\mathrm{C3}} & 0 \\ V & 0 & 0 & 0 & E_{\mathrm{C4}} \end{pmatrix} \tag{1}$$

with the main exciton transition E_{x} at 2.11 eV and the uncoupled photon mode energies E_{Cn}, as well as the coupling strength V. We achieved a quantitative agreement between experiment and theory by taking into account the four longitutinal cavity modes which are supported within the stoppband region of the Fabry-Perot cavity. The energies of the uncoupled cavity dispersions were determined by transfer matrix calculations using a background refractive index of 1.51 for the spin-coated mCherry thin film.

In order to extract the excitonic and photonic fractions of the arrising exciton-polaritons, we

determined the Hopfield coefficients n by using the following equation

$$\begin{pmatrix} E_{\text{x}} & V & V & V & V \\ V & E_{\text{C1}} & 0 & 0 & 0 \\ V & 0 & E_{\text{C2}} & 0 & 0 \\ V & 0 & 0 & E_{\text{C3}} & 0 \\ V & 0 & 0 & 0 & E_{\text{C4}} \end{pmatrix} \begin{pmatrix} \alpha \\ \beta 1 \\ \beta 2 \\ \beta 3 \\ \beta 4 \end{pmatrix} = E_{\text{Pn}} \begin{pmatrix} \alpha \\ \beta 1 \\ \beta 2 \\ \beta 3 \\ \beta 4 \end{pmatrix} \tag{2}$$

E_{Pn} are the eigenvalues of the matrix. The squares of the entries of the eigenvectors (α, $\beta 1$, $\beta 2$, $\beta 3$ and $\beta 4$) are the exciton/photon content for each polariton eigenstate.

Supplementary Figure 1 shows the false-color luminescence of a planar area next to the lattices. To highlight the upper polariton, the intensity of the energy range between 2.08 eV and 2.26 eV is magnified by a factor of 8 compared to the lower part. The positions and the curvatures of the polariton dispersions (LP1, LP2 and UP) can be reproduced by iteratively calculating the uncoupled photon modes inside the cavity using the transfer matrix method (C1, C2, C3 and C4) and fitting the intensity distribution with a coupled oscillator model. Here, a exciton energy (X) of 2.11 eV was used which was determined by reference measurements. From the fit we can estimate the optical cavity length of 2098 nm as well as a coupling strength of $V = 140$ meV (corresponding to a Rabi Energy of $\hbar\Omega_R = 2V = 280$ meV).

We furthermore extract the following system parameters for our structure: The energy of the upper polariton mode (UP) is 2.163 eV at k = 0. The energy of the two lower polariton resonances

in the spectral vicinity of the exciton are 1.970 eV (LP1) and 1.778 eV (LP2).

From the determined coupling strength and energies, for the mode LP1 we can calculate the excitonic fraction 16.1 %, photonic fraction 83.9 %, and for the mode LP2 we yield an excitonic fraction 8.3 %, photonic fraction 91.7 %.

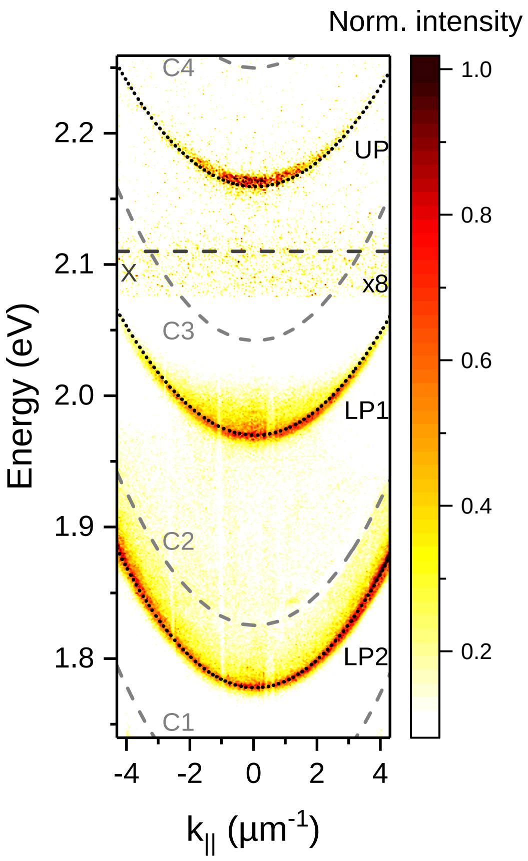

Supplementary Figure 1: False-color PL spectrum of a planar area next to the lattices. The spectrum is composed of two lower (LP1, LP2) and an upper (UP, maginified by a factor of 8) polariton branch. The polariton dispersions can be reproduced using a coupled oscillator model (black dotted lines) with a coupling strength of $V = 140\,\text{meV}$.

Supplementary Note 2 ”Information on the one-dimensional lattice”

Our one dimensional lattices are composed of linear chains with 40 sites, yielding an overall length of 100 µm. Our sample features two main areas with nominally identical chains, which feature a slightly different detuning. This is a consequence of a natural wedge in the sample, which occurs during the mechanical assembly.

The structure, which is investigated in detail in Fig. 2 (main text and corresponding passage) features a polariton energy (k = 0) of 1.95 eV. This yields an optical cavity length about 2140 nm, and an excitonic content of approx. $16\ \%$. The energy is slightly red-shifted with respect to the planar mode LP1 introduced in S1 due to the hemispheric elongation and the redshift is partially compensated by the optical confinement of the lattice. The device, which is investigated in Fig. 3 of main text (and the corresponding passage) features a polariton energy of 1.87 eV. This yields an optical cavity length 2230 ± 15 nm, and reflects an excitonic contribution of approximately $15\ \%$.

Supplementary Note 3 ”Assessment of photonic coupling in the one-dimensional lattice”

Photonic coupling in our one-dimensional lattices is clearly dictated by the overlap of neighboring traps. While in Fig. 1 of the main text, we discuss the evolution of the miniband structure from a single trap, via the molecular coupling to the full lattice, here, we depict the evolution of the lattice band structure as we modify the next neighbor coupling. All traps have a size of 5 µm, and the next-neighbor distance is varied from Supplementary Figure 2 b) to d) from 3 to 2.5 µm. Supplementary Figure 2 a) depicts the spectrum of a single, isolated trap as a reference. Clearly,

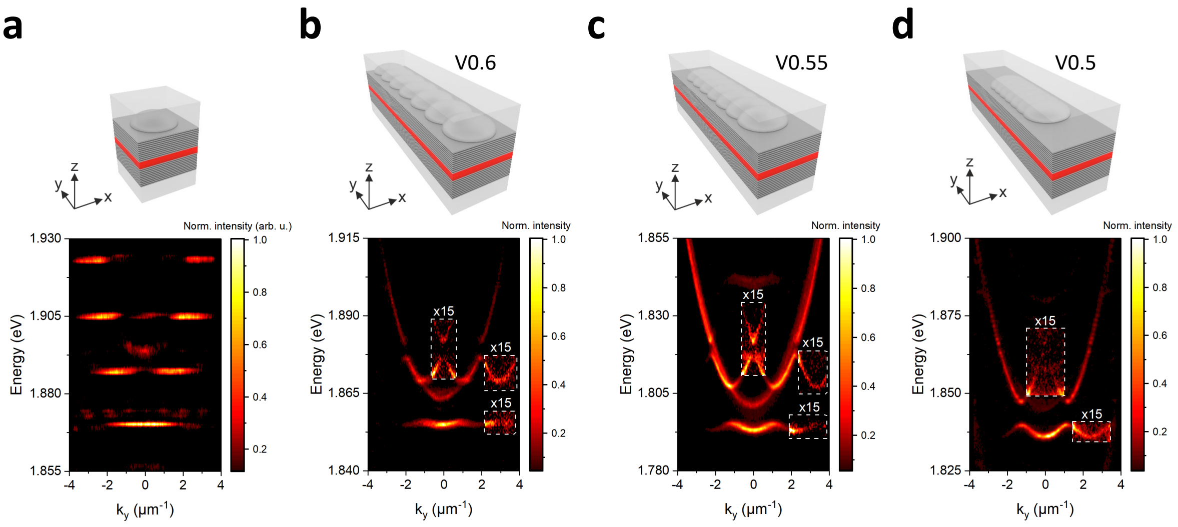

Supplementary Figure 2: Evolution of the photonic band-structure for one dimensional lattices with varying next-neighbor coupling. The overlap between neighboring lattice sites (traps) is systematically increased from b) to d), resulting in a clear increase of curvature and bandwidth in the s-band, as well as a strongly increased bandwidth of the p-band. Panel a) depicts the far-field emission spectrum of a single trap as a reference.

upon increasing the overlap between neighboring traps, the evolution of the bandwidth, both in the s- as well as the p-band becomes evident.

Supplementary Note 4 ”Input-Output characteristics of the one-dimensional polariton lattice”

In Fig. 2e (main text), we present and discuss the power-dependent behavior of the one-dimensional lattice. While the input-output characteristics is depicted in double logarithmic style in the main text, in Supplementary Figure 3, we plot the data in linear style. In both cases, a clear threshold

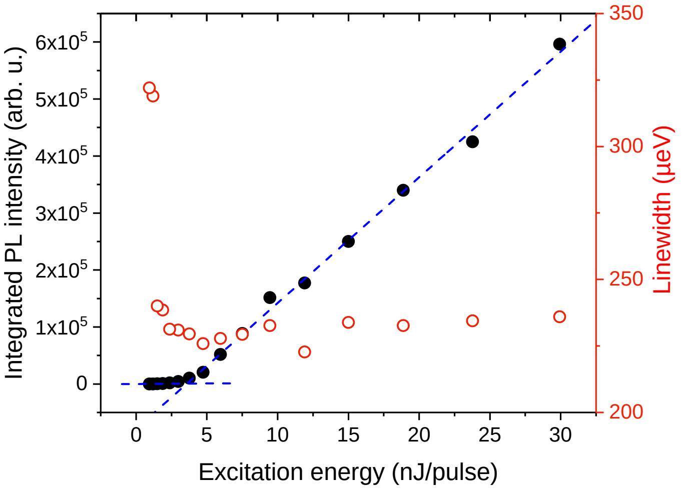

Supplementary Figure 3: Linear plot of the Input-output characteristics, as well as the linewidth of the polariton mode of the lattice which is discussed in Fig. 2e of the main text.

behavior can be captured.

Supplementary Note 5 ”Numerical modeling”

For numerical calculations of the polariton dynamics in organic chains we consider a two-dimensional mean-field model, which has been widely used for simulation of inorganic semiconductor cavities [1,2] and very recently for organic systems [3]. This model consists of the open-dissipative Gross-Pitaevskii (GP) equation for the condensate wave-function and coupled to the rate equation for the excitonic reservoir created by the off-resonant pump [2,4]. Above condensation threshold a coherent part of condensate, given by the order parameter ψ, is described by the GP-type equation and the exciton reservoir density n_r by the rate equation [1]

$$i\hbar\frac{d\psi}{dt} = \left(-\frac{\hbar^2}{2m}\nabla^2{}_{x,y} + V(x,y) + g_c|\psi|^2 - \frac{i\hbar}{2}\gamma_c + \left(g_r + \frac{i\hbar R}{2}\right)n_r\right)\psi + i\hbar\frac{d\psi_{st}}{dt}, \tag{3}$$

$$\frac{dn_r}{dt} = -\left(\gamma_r + R|\psi|^2\right) n_r + P(x,y). \tag{4}$$

An external optical beam of Gaussian shape $P(x,y) = P_0 e^{-(x^2+y^2)/s^2}$ (with the radius s) populates the reservoir of incoherent excitons. The reservoir induces a net gain given by the term $i\hbar R n_r/2$, where $R = 1.5 \cdot 10^{-6}$ ps^{-1} μm^2 defines the condensation rate. Constants $\gamma_c = 0.45$ ps^{-1} and $\gamma_r = 0.15$ ps^{-1} represent the decay rates of polaritons and reservoir excitons, respectively. While the polariton damping constant γ_c can be directly estimated from the line width, the choice of the condensation R and the reservoir damping γ_r rates are more sophisticated. Our choice of these parameters was mostly governed by a correct prediction of the pumping rate at the condensation threshold known from the experiment (see also discussions below).

The scattering of the particles from reservoir (with the rate $\hbar R n_r/2$) as well as the damping γ_c induce stochastic fluctuations of the condensate, which are accounted for by the stochastic term $d\psi_{st}(\mathbf{r}_i) = \sqrt{\frac{\gamma_c + R n_r(\mathbf{r}_i)}{4\delta x \delta y}} dW_i$ [2,6]. Here dW_i is a Gaussian random variable characterized by the correlation functions $< dW^*{}_i dW_j >= 2\delta_{i,j} dt$ and $< dW_i dW_j >= 0$ where i,j are indices counting discrete mesh points $\mathbf{r}_i$ with the discretizations δx and δy in x and y directions, respectively.

The condensate experiences a blue shift induced by repulsive interaction between polaritons as well as due to elastic scattering at the reservoir particles, represented by the terms $g_c|\psi|^2$ and the term $g_r n_r$, respectively. Constants $g_c = 0.5 \times 10^{-6}$ meV μm^2 and $g_r = 3.2 g_c$ characterize the strengths of polariton-polariton and polariton-reservoir interactions, respectively. These parameters were estimated from the experimentally obtained photoluminescence spectra recorded at various pump powers (see Fig. 2 of the main text).

The kinetic energy of polaritons is characterized by the effective mass, m, which was taken as $\times 6.96 \cdot 10^{-6}$ of the free electron mass. The periodic array with the period $a = 2.5$ μm is modeled by the two-dimensional potential $V(x, y)$ and consists of spatially-overlapping mesa traps. A separate mesa has a half-ellipse potential profile defined as the real part of the function: $V(x, y) = V_0\sqrt{1 - 4(x^2 + y^2)/d^2}$ with the diameter $d = 5$ μm and the potential depth $V_0 = -270$ meV.

The nonequilibrium Gross-Pitaevskii approach (3,4) with additional stochastic terms allows for a modeling of the experimental observations for pumping rates below and above the condensation threshold. Here we consider an example of on-site excitation of the condensate and formation of the localized condensed state in energy gap just above the first band of the single-particle band-gap spectrum. Below the condensation threshold, Bloch bands are visible in the spectrum of lower polaritons, as shown in Supplementary Figure 4(a). We mention that the calculated linear band-gap spectrum of the polaritons coincides almost exactly with the experimental data, justifying the choice of the model parameters describing the kinetic properties of polaritons (the effective mass m) and the potential shape $V(x, y)$.

When excited above threshold by a narrow pump focused on a potential minimum (a trap site), the condensed state of exciton-polaritons forms in the gap, as demonstrated in Supplementary Figure 4 (b) and (c). This state has maximum emission at the edges of the Brillouin zone (BZ) ($k = \pm\pi/a$) and, thus, its phase profile inherits the characteristic π-phase shift between two neighbouring sites Supplementary Figure 4(f). Note that the reservoir-induced blue shift prevents

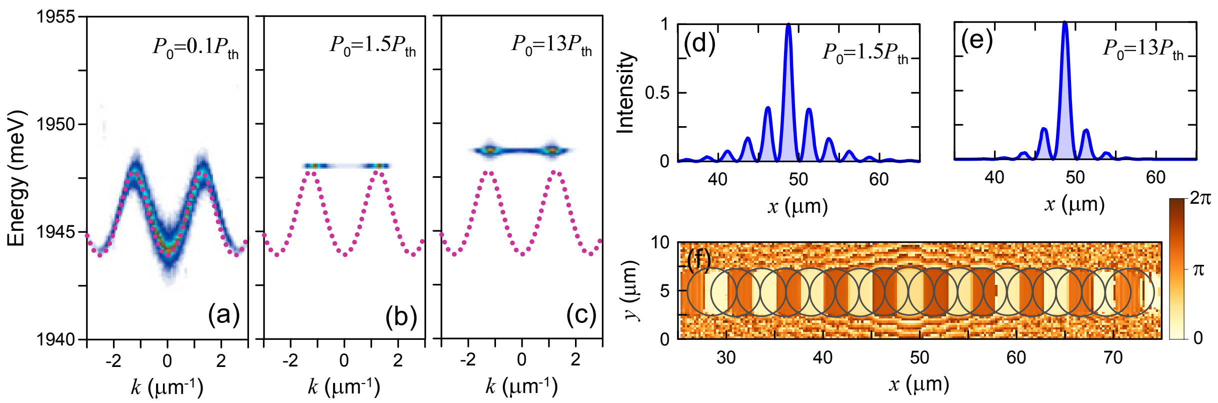

Supplementary Figure 4: Polariton population (emission intensity) under on-site incoherent excitation of the organic chain calculated for different pumping rates. The spectra are recorded along the line connecting the traps' centers (i.e. for $y = 0$). (a) Below condensation threshold $P_0 = 0.1P_{\text{th}}$, where the condensation threshold for the chain is $P_{\text{th}} \approx 165 \cdot 10^3$ μm^{-2} ps^{-1}. (b) In the vicinity of the condensation threshold $P_0 = 1.5P_{\text{th}}$. (c) Far above threshold $P_0 = 13P_{\text{th}}$. The dashed red lines show calculated linear wave dispersion of the chain. The panels (d) and (e) show the spatial intensity profiles (normalized to the maximum) in the vicinity [corresponds to (b)] and far above [corresponds to(c)] condensation threshold, respectively. (f) The phase of the state given on panel (d). For the modeling we used $s = 1.1$ μm which corresponds to the pump spot diameter (FWHW) $d_{\text{FWHW}} = 1.83$ μm.

condensation into the single-particle band at least for the given set of parameters. Furthermore, in the vicinity of the threshold, spatial extension of the condensate is very sensitive to the polariton-reservoir interaction constant g_r, which affects directly the off-set of the condensate energy from the band edge.

For even stronger pumping, the condensate experiences further blue-shift into the gap due to the repulsive polariton-polariton interaction Supplementary Figure 4(c). As a results, the spatial extensions of the condensate profiles becomes narrower for the increasing pumping rates, as shown in Supplementary Figure 4 (d) and (e). This gap states survives over the wide interval of pump powers exceeding up to 20 times the threshold value.

To conclude this section we mention that the pumping rate for the condensation threshold was numerically found to be about $P_{\text{th}} \approx 165 \cdot 10^3$ µm^{-2} ps^{-1}. Assuming the pumping pulse duration of 7 ns and the spot diameter of about 1.83 µm (used for calculations), it gives the value 1.14 nJ/pulse which is a very reasonable estimation of the condensation threshold known from the experiment (1.5 nJ/pulse).

Supplementary Note 6 ”Far-field spectra of the localized gap-state”

Far-field spectra of the localized gap state under on-site excitation are shown in Supplementary Figure 5. The spectra correspond to the measurements presented in Fig. 4 of the main text. A clear shift of the mode inside the bandgap above the polariton condensation threshold is clearly evident. The white line shows the calculated of a single-particle eigenstates s-band in the effective periodic

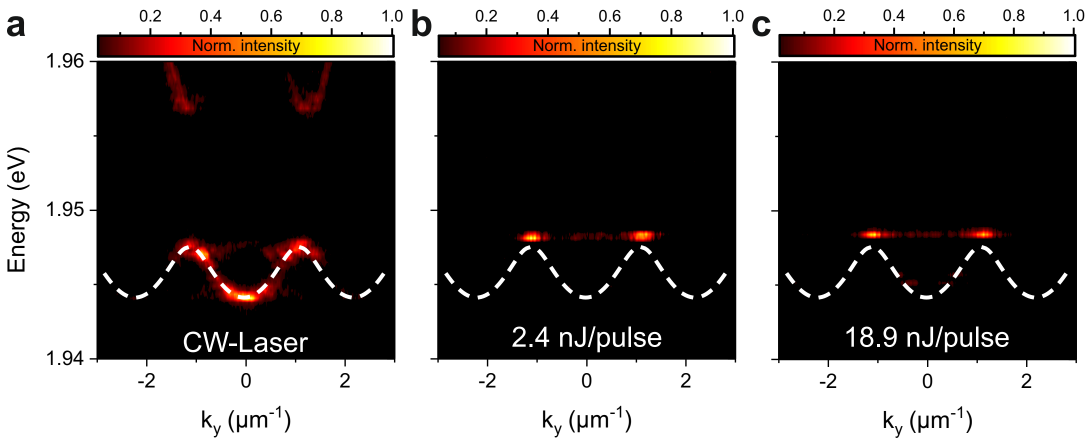

Supplementary Figure 5: Far-field spectra of the localized gap state under on-site excitation (corresponding to Fig. 4 in the main text). The shift of the polariton mode inside the forbidden bandgap for increasing excitation densities is clearly evident.

potential.

Supplementary Note 7 ”Expansion of the s-band condensate”

In Fig. 4 of the main manuscript, we present data revealing the localization of a condensate in a device which was injected under on-site excitation conditions. The condensate formed on top of the s-band, and shifted into the gap. In contrast, Supplementary Figure 6 summarizes the overall behavior of a device with a very similar under off-site conditions. There, the condensate forms at the lowest energy state of the s-band, and expands in real space as the pump is increased. Just above threshold (Supplementary Figure 6 f), the condensate has an approximate expansion of 6.2 μm, whereas the expansion increases to approx. 9 μm for a pump energy of 18.9 nJ (Supplementary Figure 6 g). This is in stark contrast to behaviour of our condensates, which are prepared on the

high energy side of the first miniband, which has been analysed in Fig. 4 of the manuscript. There, the real-space expansion reduced with pump power from 7 μm to 3.6 μm.

Supplementary Note 8 ”Long range coherence in the lattice”

In order to assess the coherence, as well as the expansion of the condensate in our lattice, we utilize the interferometric setup depicted in Supplementary Figure 7 a). The real-space image of the photoluminescence signal of the condensate is brought to overlapp with its mirror-image (generated via the retro-reflector) on a high-resolution CCD camera, and the corresponding interference image is analyzed. While, in Fig. 3 of the main text, we discuss first order coherence arrising in the condensed state on top of the s-band, in order to generate an expanding condensate, we deliberately load the condensate into the p-band by vertically offsetting the pump (see Supplementary Figure 7 b). The according interference pattern in Supplementary Figure 7 c) exhibits significant fringes, characterizing a coherent mode. By varying the tuneable delay in the interferometer, one can directly assess and quantify the phase of the coherent state, plotted in Supplementary Figure 7 d) and e).

We note, that the high quality of our sample allows us to observe a significant coherent fraction of the mode expanding over more than 10 lattice sites (Supplementary Figure 7 d). Furthermore, we observe the characteristic Pi shift between neighboring lattice sites in Supplementary Figure 7 e).

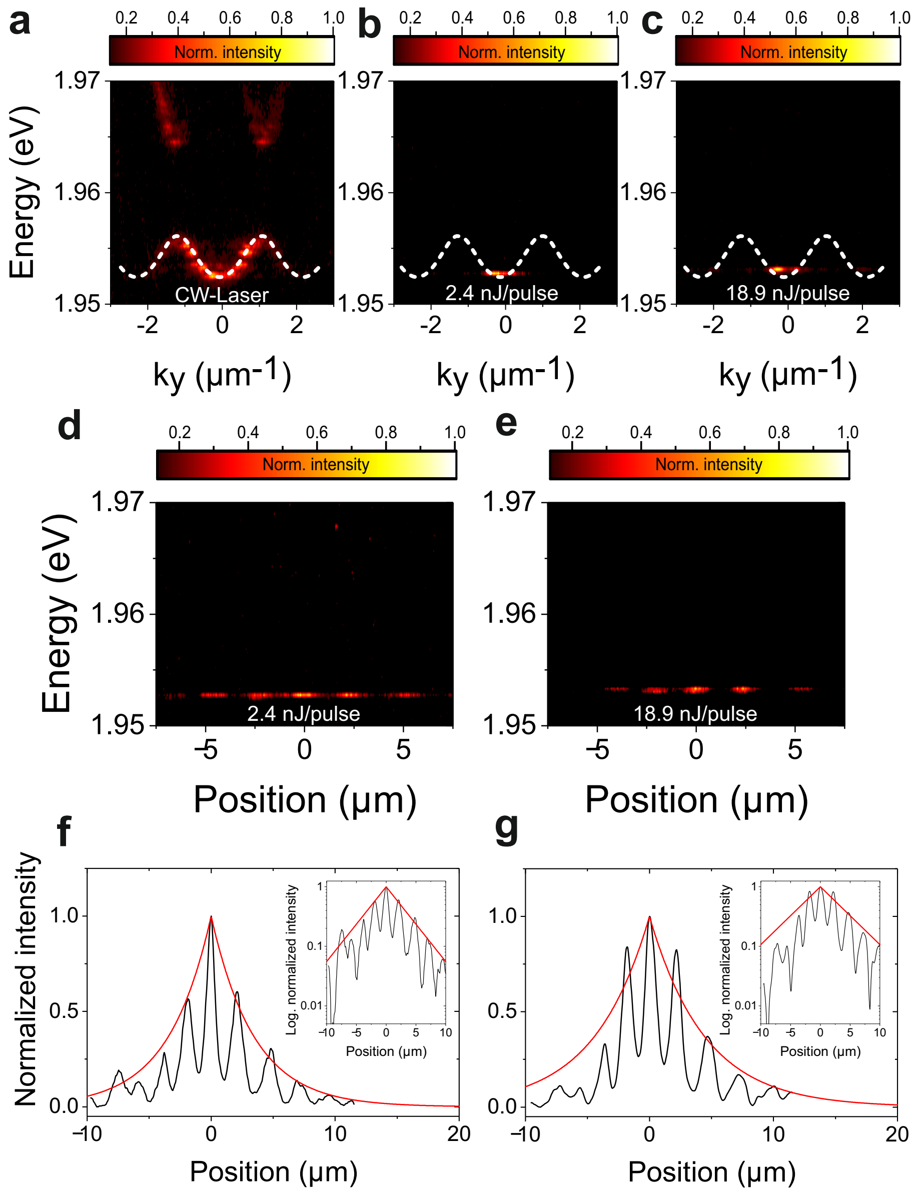

Supplementary Figure 6: **a, b, c** Far-field spectra of a selected device recorded under off-site excitation. Condensation in the low energy state of the s-band occurs at elevated pump powers. **d, e,** Energy-resolved realspace images under off-site pumping conditions little above threshold (**d**, P = 2.4 nJ/pulse), and far above threshold (**e**, P = 18.9 nJ/pulse). **f, g,** The intensity traces are fitted by an exponential function. In the insets, the same profiles are shown in log scale.

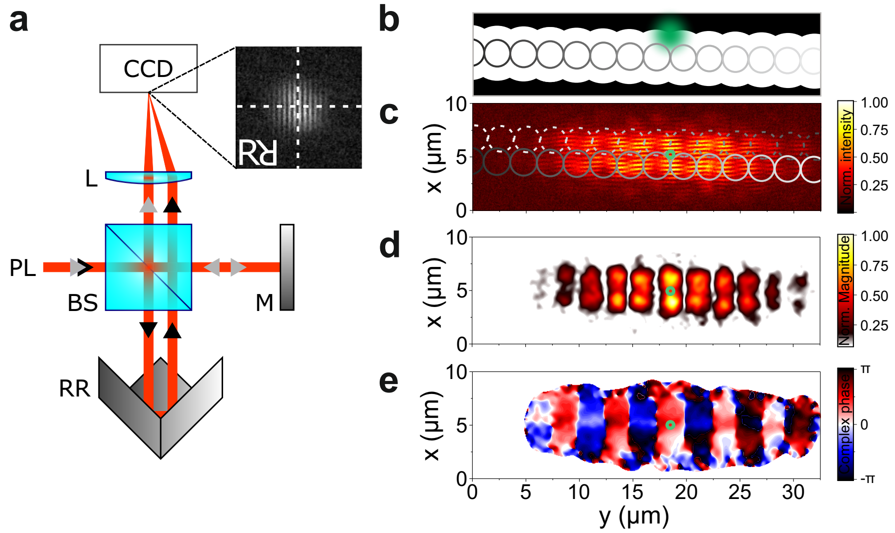

Supplementary Figure 7: a) Schematic drawing of the Michelson interferometer utilized to measure the coherent fraction of the polariton condensate formed in the lattice. b) Sketch of the pumping conditions chosen in the reference experiment, assisting to load the condensate into the p-band. c) Interference pattern recorded via the setting in a), by slightly offsetting the signal from the retro-reflector. d) Coherent fraction, and e) phase of the condensate as a function of position in the lattice. The centro-symmetric point is indicated in c-e)

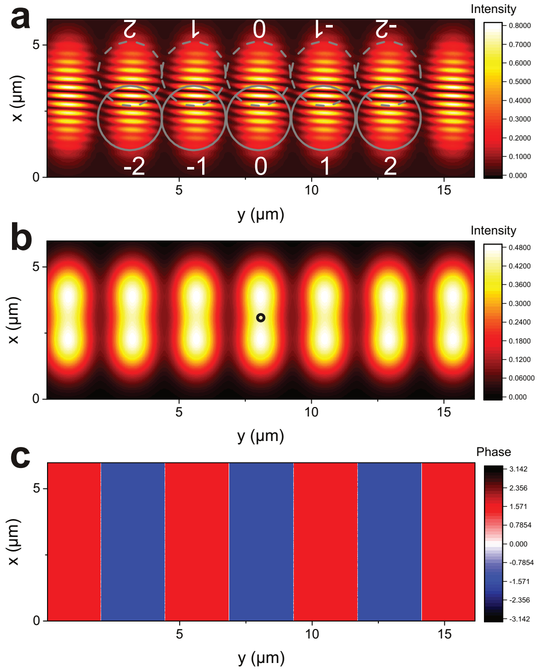

Supplementary Figure 8: (a) Calculated interference pattern. The solid (dashed) circles indicate the positions of the different sites of the linear chain (the double mirrored linear chain). A shift as well as a tilt was introduced to match the condition of the experiment. (b) Square of the absolute value of the E-field and (c) phase of the E-fields as a function of the position neglecting the tilt between the two images. The black circle in (b) indicates the centro-symmetric point.

Supplementary Note 9 ”Data analysis arising from interferometric measurements”

Supplementary Figure 8 depicts a calculated interference pattern for a comparable setting as introduced in the experiment depicted in Fig 3 (c-e) of the main text. The calculation evidences the feasability of our approach to utilize a vertically shifted, mirrored image to study the self-interference.

Supplementary References

1. Wouters, M. & Carusotto, I. Excitations in a nonequilibrium bose-einstein condensate of exciton polaritons. *Phys. Rev. Lett.* **99**, 140402 (2007).

2. Wouters, M., Carusotto, I. & Ciuti, C. Spatial and spectral shape of inhomogeneous nonequilibrium exciton-polariton condensates. *Phys. Rev. B* **77**, 115340 (2008).

3. Bobrovska, N., Matuszewski, M., Daskalakis, K. S., Maier, S. A. & Kèna-Cohen, S. Dynamical instability of a nonequilibrium exciton-polariton condensate *ACS Photonics* **5**, 111 (2018).

4. Winkler, K. et al. Collective state transitions of exciton-polaritons loaded into a periodic potential. *Phys. Rev. B* **93**, 121303(R) (2016).

5. Betzold, S. et al. Coherence and interaction in confined room–temperature polariton condensates with frenkel excitons. *ACS Photonics* **7**, 384 (2020).

6. Wouters, M. & Savona, V. Stochastic classical field model for polariton condensates. *Phys. Rev. B* **79**, 165302 (2009).
